# Down regulation of E-Cadherin (ECAD) - a predictor for occult metastatic disease in sentinel node biopsy of early squamous cell carcinomas of the oral cavity and oropharynx

**DOI:** 10.1186/1471-2407-11-217

**Published:** 2011-06-03

**Authors:** Gerhard F Huber, Lena Züllig, Alex Soltermann, Matthias Roessle, Nicole Graf, Stephan K Haerle, Gabriela Studer, Wolfram Jochum, Holger Moch, Sandro J Stoeckli

**Affiliations:** 1 Otorhinolaryngology, Head and Neck Surgery, University Hospital Zurich, Switzerland; 2 Institute of Surgical Pathology, University Hospital Zurich, Zurich, Switzerland; 3 Clinical Trials Center, Center for Clinical Research, University Hospital Zurich, Switzerland; 4 Department of Radiation Oncology, University Hospital Zurich, Zurich, Switzerland; 5 Department of Pathology, Kantonsspital St.Gallen, Switzerland; 6 Otorhinolaryngology, Head and Neck Surgery, Kantonsspital St.Gallen, Switzerland

**Keywords:** Head and Neck squamous cell carcinoma (HNSCC), oral cavity, oropharynx, E-Cadherin (ECAD), Immunohistochemistry, Sentinel node biopsy

## Abstract

**Background:**

Prognostic factors in predicting occult lymph node metastasis in patients with head and neck squamous-cell carcinoma (HNSCC) are necessary to improve the results of the sentinel lymph node procedure in this tumour type. The E-Cadherin glycoprotein is an intercellular adhesion molecule in epithelial cells, which plays an important role in establishing and maintaining intercellular connections.

**Objectives:**

To determine the value of the molecular marker E-Cadherin in predicting regional metastatic disease.

**Methods:**

E-Cadherin expression in tumour tissue of 120 patients with HNSCC of the oral cavity and oropharynx were evaluated using the tissue microarray technique. 110 tumours were located in the oral cavity (91.7%; mostly tongue), 10 tumours in the oropharynx (8.3%). Intensity of E-Cadherin expression was quantified by the Intensity Reactivity Score (IRS). These results were correlated with the lymph node status of biopsied sentinel lymph nodes. Univariate and multivariate analysis was used to determine statistical significance.

**Results:**

pT-stage, gender, tumour side and location did not correlate with lymph node metastasis. Differentiation grade (*p *= 0.018) and down regulation of E-Cadherin expression significantly correlate with positive lymph node status (*p *= 0.005) in univariate and multivariate analysis.

**Conclusion:**

These data suggest that loss of E-cadherin expression is associated with increased lymhogeneous metastasis of HNSCC. E-cadherin immunohistochemistry may be used as a predictor for lymph node metastasis in squamous cell carcinoma of the oral cavity and oropharynx.

Level of evidence: 2b

## Background

Head and neck squamous cell carcinoma (HNSCC) is the fifth most common malignancy worldwide. In 1999, in the US, approximately 29'800 patients suffered from a squamous cell carcinoma of the oropharynx and the oral cavity and more than 8000 died of it [1]. Despite improvements in surgical treatment and radiation technology over the last decades, prognosis remains dismal in advanced cases. Regional metastatic disease is known to reduce recurrence free survival and disease specific survival significantly [2].

A chief feature of malignant behavior is the capability of tumour cells to metastasize. Metastatic spread is an extremely bad prognostic factor and responsible for cause of death in ~90% of all cancer patients [3]. The cellular mechanisms responsible for the acquisition of a metastatic phenotype include the adaptation to potential hostile environment (blood stream, lymph nodes, organ of metastasis). In addition, the "tumour cell-to-tumour cell" and "tumour cell-to-stromal environment" cross talk is recognized as an important condition for invasion and metastasis.

Sentinel node biopsy (SNB) for the cN0 neck in early HNSCC of the oral cavity has been validated by multiple studies. The workup of sentinel lymph nodes is performed as described elsewhere [4] and reliably detects occult metastatic disease. Further, occult metastatic disease is subdivided in macrometastasis (>2 mm), micrometastasis (0.2-2 mm) and even small tumour cells or small clusters <0.2 mm (isolated tumour cells, ITC).

The hitherto published predictive factors for metastatic disease in early HN tumours are histomorphological parameters like mode of invasion (MOI; morphological appearance of the infiltrating tumour front), depth of tumour infiltration, grade of differentiation (GOD), lymphatic invasion (LI) [5] and intratumoural lymphatic density [6].

The ECAD glycoprotein (encoded by the *CDH1 *gene, located on chromosome 16q22.1) is a Ca2^+^-dependent intercellular adhesion molecule in epithelial cells, which plays an important role in establishing and maintaining intercellular connections and morphogenesis. The cytoplasmatic terminus of the ECAD molecule has been shown to be linked to the actin cytoskeleton via α-catenin and β-catenin [7].

Dysfunction of ECAD/catenin complex is directly involved in carcinogenesis. *CDH1 *is considered to be a tumour suppressor gene, whose loss has also been demonstrated to promote tumour invasion and metastasis in various cancer models [8]. There are several mechanisms for abnormal ECAD expression in cancer, including allelic loss at the *CDH1 *locus as well as somatic and, rarely, germ line mutations. Transcriptional repression of ECAD by promoter hypermethylation has also been reported in several tumour types and cell lines and reduced expression of ECAD in esophageal Adenocarcinoma was shown to correlate with poor prognosis [9,10].

Changes or alterations in the function and expression of this cell to cell adhesion molecule have been postulated to be an early event in the multiple step process of tumour metastasis and an important factor in tumour progression [10].

It is reported that down regulation of α-catenin and β-catenin seems to be associated with dysfunction of ECAD mediated cell adhesion and an increase in the metastatic potential of cancer cells [11].

The correlation between the expression of the ECAD-associated molecules and the presence of neck metastasis is significant, indicating that reduced expression of ECAD is a key function in the increased incidence of neck metastasis [12].

In this study, we aimed to determine the potential of ECAD expression in predicting early metastatic disease of patients with HNSCC of the oral cavity and oropharynx by analyzing a very well defined patient cohort (T1,T2 oral cavity and oropharyngeal cancers) with meticulous workup of sentinel lymph nodes.

The hypothesis was that low expression of ECAD leads to reduced tumour cell to tumour cell adhesion and therefore easier disintegration of single tumour cells with increased risk of occult metastatic disease in analogy to the results published by Hirata et al. for small cell lung cancer [12].

## Methods

### Patients

Study permission was obtained by the local ethical board commission of Zurich.

We retrospectively analyzed formalin-fixed, paraffin-embedded primary tumour tissues of 120 patients for ECAD expression. Thirteen patients had been excluded in advance because of insufficient material on the paraffin block of the primary tumour or lost follow up.

A majority of the patients in this study were participants in a prospective clinical trial of SNB of which the results were already published [4].

In order to detect ECAD expression differences of the primary tumour and the node metastases, we planned to examine the corresponding lymph nodes of all patients with occult metastatic disease (n = 45). Unfortunately only in 10 patients there was enough material of the sentinel lymph nodes to be analyzed at the same time with the primary tumour, because no remaining material was left for further immunohistochemistry.

Hundred-eleven patients were diagnosed and treated in the ENT department of the University Hospital of Zurich, nine at the Kantonsspital St. Gallen, Switzerland.

The ENT-clinic of the University Hospital Zurich is one of the places with the largest single institution experience in sentinel node biopsy for head and neck cancers. Since the radioactive tracer is injected not intraoperatively but in the awake patient, only tumours of the oral cavity and the anterior oropharynx can be accessed safely by the physician and without relevant patient discomfort. We were aware that especially tumours of the tonsillar pillar behave differently compared to their oral cavity counterparts (HPV-Status); however, we only included patients in the study with "anterior oropharyngeal cancers" which encompasses cancers localized on the anterior palatal arch and therefore most likely behave more like an oral cavity tumour than a HPV-positive tonsillar carcinoma.

**Figure 1:**
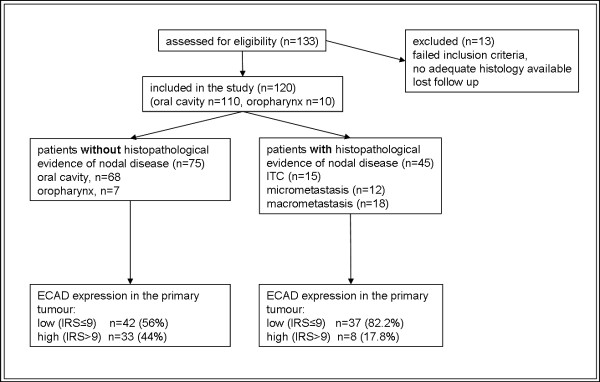
**Consort Diagram of the patients included in the study**. ECAD = E-Cadherin; IRS = Intensity Reactivity score; ITC = isolated tumour cells

All patients were staged according to the guidelines of the Swiss head and neck working group which corresponds to the AJCC/UICC staging manual (7^th ^edition 2009). A detailed history was taken with special attention given to the exposure or use of tobacco and/or alcohol. Clinical examination was amended with chest radiograph, electrocardiogram, computed tomography (CT) and/or magnetic resonance imaging (MRI) of the head and neck. Additionally, every patient was examined by ultrasound and fine needle aspiration cytology (FNAC) in case of suspicious lymph nodes. In case of positive nodal disease, patients were treated with selective neck dissection according to the primary tumour locations or modified radical neck dissection and thus excluded from the sentinel node biopsy study. A flow diagram that illustrates the patients included and excluded from the study is depicted in figure 1.

Inclusion criteria:   - Patients with early stage (T1/T2) SCC of the oral cavity or the oropharynx and clinical and radiological negative regional disease (cN0)

                             - Patients who received lymphoscintigraphy and intraoperative sentinel node dissection

Exclusion criteria:   - Patients with advanced stage (T3/T4) SCC of the oral cavity or oropharynx and clinical and radiological negative regional disease (cN0) or early stage primary tumour and clinically positive neck disease (cN+)

                             - Patients who were treated by radio-chemotherapy and not surgically

Clinical follow-up was available in all 120 patients (mean clinical follow up 81 months; range 11-122 months).

### Sentinel lymph node biopsy

The sentinel lymph node biopsy (SNB) procedure and pathological work up of SLN has been recently reported [4,13]. In brief, SNB was performed according to the following standardized protocol: The lymph node mapping consisted of a preoperative lymphoscintigraphy and the intraoperative use of a hand-held gammaprobe. The location of the SLN was marked on the patient's skin under radiologic guidance. As the first surgical step, the primary tumour was removed using a transoral approach. For guidance to the SLN, two different hand-held gammaprobes with collimated 14 mm tips were used (Navigator GPS, Tyco Health Care Switzerland Ltd, Wollerau, Switzerland and Neo 2000, Neoprobe Corporation, Dublin, OH). The skin markings were confirmed with the gammaprobe and a small skin incision of 3 to 5 cm was made in a location that could be extended for subsequent neck dissection. With the guidance of the gammaprobe, the SLN was identified by blunt dissection. In most cases, more than one SLN was present. All the SLNs were excised selectively and the activity level was measured *ex situ*. Lymph nodes were considered to be SLNs if their activity counts were at least three times the count of the background. In cases where a subsequent neck dissection followed, the neck dissection specimen was searched with the gammaprobe for missed SLNs. Elective neck dissection comprised levels I to III for oral cavity cancer and levels II to IV for oropharyngeal cancer. SLNs were assessed intraoperatively with frozen section analysis. In case of detection of occult metastases, an immediate elective neck dissection encompassing the above-mentioned levels was performed in the same surgical session. For frozen sections, each SLN was bisected and a fine slice of the surface of each half was stained with hematoxylin and eosin (H&E). The remainder of the SLNs were thawed immediately and paraffin-embedded for complete histopathologic evaluation. Extensive histopathologic work-up of all SLNs and every node of the neck dissection specimen was performed according to the same standardized protocol. It is well known that the more precisely the lymph nodes from a neck dissection specimen are examined, the more metastases will be found [14]. Therefore, a very extensive histopathologic work-up of all nodes was performed to achieve the highest possible accuracy of results. The lymph nodes were first lamellated manually into slices of 2 mm, and each slice was step sectioned with the microtome at intervals of 150 μm. Each slice was stained with H&E and cytokeratin for immunohistochemistry. The presence of occult metastases was assessed and their size measured with a standardized measuring ocular device. If no cytokeratin positivity was found, the node was declared tumour free. If cytokeratin deposits were detected, the positive section was compared with the immediately adjacent serial section previously stained with H&E to determine whether the positivity was due to the presence of viable tumour cells.

### Tissue microarray construction

Paraffin-embedded tissue of 120 tumours was used for the preparation of tissue microarray. A morphologically representative region of the paraffin "donor" blocks was chosen. From the representative region two core tissue biopsies (diameter, 0.6 mm; height, 3-4 mm) from the invading front were taken and precisely arrayed into a new "recipient" paraffin block using a custom-built instrument [15]. After the block construction was completed, 4.0-μm sections of the resulting tumour tissue microarray block were cut for further analysis.

### Immunohistochemistry

ECAD immunohistochemistry was performed on paraffin sections of formalin-fixed tissues, using the Cadherin-E antibody (1:200, Clone EP700Y, Cell Marque Lifescreen Ltd.) and a Ventana Benchmark automated staining system (Ventana Medical Systems) as recently described [16,17].

Staining intensity was assessed using the ***I***ntensity ***R***eactivity ***S***core (IRS), where staining intensity (SI) was assessed to be negative (= 0), weak (= 1), moderate (= 2) or strong (= 3) and reactivity was determined by percentage of positive cells (PP) where negative specimens were 0, 1-10% of cell (= 1), 11-30% (= 2), 31-50% (= 3), 51-80% (= 4) and >80% (= 5). IRS was calculated by multiplying SI with PP resulting in a minimum of 0 and a maximum of 15. Besides IRS, expression patterns were assessed. Per patient, two samples were stained and average IRS was taken for statistical analysis.

Tumours were categorized into ECAD low (IRS = 0-4), moderate (IRS = 5-9) and high (IRS10-15).

### Statistics

The relationship between ECAD expression and presence of lymph node metastasis was analyzed using Fisher's exact test. Subsequently, a logistic regression was performed to evaluate the predictive power of ECAD expression for lymph node metastasis while controlling for differentiation grade and tumour stage. Unfortunately, tumour differentiation grade could not be included as a factor in the logistic regression model because of quasi-complete separation in the data. The potential usefulness of ECAD expression as a predictive marker for lymph node metastasis was further specified with details about sensitivity and specificity. True positive was defined as [number of individuals with low expression/individuals with positive sentinel lymph nodes]. False positive was defined as [number of individuals with low expression/individuals with negative lymph nodes]. True negative and false negative was defined accordingly (table 1).

**Table 1:** Calculation of Sensitivity and Specificity depending on ECAD expression ("2x2 table").

		"gold standard" = result of sentinel node biopsy		
		**occult metastatic disease: Yes**	**occult metastatic disease: No**	

**new predictive marker for occult metastatic disease (ECAD)**	**Low **ECAD	true positive	false positive	all test positives
	expression	**a**	**b**	**a + b**
	**High **ECAD	**c**	**d**	all test
	expression	false negative	true negative	negatives
				**c + d**

		**a + c**	**b + d**	
		all with occult	all without occult	
		disease	disease	

Hypothesis: low ECAD expression (test) predicts occult metastatic disease

sensitivity: a/(a + c)

specificity: d/(b + d)

Finally, a Kaplan-Meier survival analysis with calculation of log rank statistics was performed to compare survival and relapse rates, respectively, between patients with high and low expression. PASW Statistics 18.0.0 for windows (SPSS) was used for statistical analyses.

## Results

Mean patient age was 59 years (range 28-88). The tumours were located in the oral cavity (110/91.7%; mostly tongue) and the oropharynx (10/8.3%). Male: Female ratio was 82:38 (2.16:1). 63 patients presented with a T1 carcinoma, 55 with a T2 and in two cases T stage was not clearly defined. Both sides (right vs. left) were involved equally (46.7% vs. 42.5% respectively). In addition, 10.8% of tumours were located in the midline.

T-stage (p = 0.252), gender (p = 1.000), tumour side (p = 0.926) and location (p = 1.000) were not found to correlate with positive lymph node status. Table 2 summarizes the results of the clinical parameters.

**Table 2:** Association of E-cadherin with tumour stage, differentiation grade and lymph node status

	Cadherin low (IRS ≤ 9) n = 79 (65.8%)	Cadherin high (IRS > 9) n = 41 (34.2%)	*P*-value
**Tumor stage**			0.445^1)^
pT1	40 (50.6%)	24 (58.5%)	
pT2	39 (49.4%)	17 (41.5%)	
Differentiation grade*			0.018^2)^
Grade I (well)	7 (9.5%)	7 (18.4%)	
Grade II (moderate)	51 (68.9%)	29 (76.3%)	
Grade III (poor)	16 (21.6%)	2 (5.3%)	
**Sentinel node**			0.005^1)^
negative	42 (53.2%)	33 (80.5%)	
positive	37 (46.8%)	8 (19.5%)	

*Eight patients with differentiation grade not otherwise specified have been excluded

^1) ^Fisher's exact test, ^2) ^exact Mann-Whithey U test

**Figure 2:**
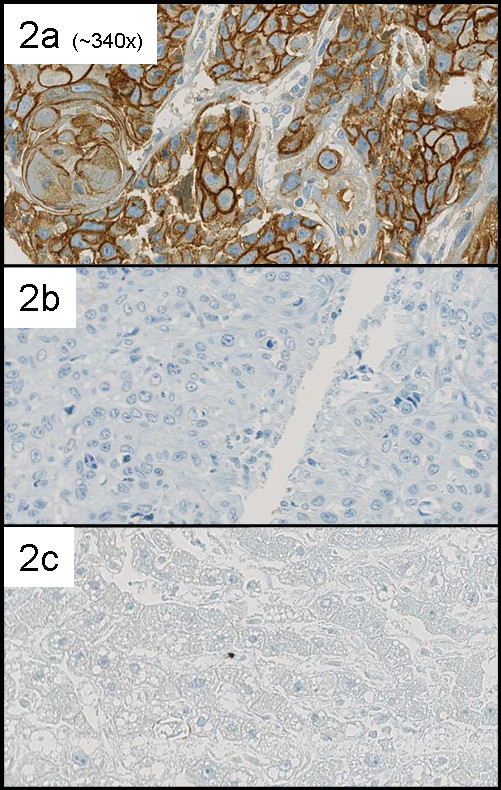
**2a strong, mainly membaneous ECAD expression; 2b absent ECAD expression in another tumour; 2c negative control**.

Immunohistochemical ECAD expression of tumour cells was predominantly membranous (figure 2). Some undifferentiated SCC's also showed cytoplasmatic staining. Forty-one of 120 (34.2%) of the primary tumours were ECAD high. High ECAD expression was associated with pathologic grade (table 2). No association between ECAD expression and tumour stage was observed.

**Figure 3:**
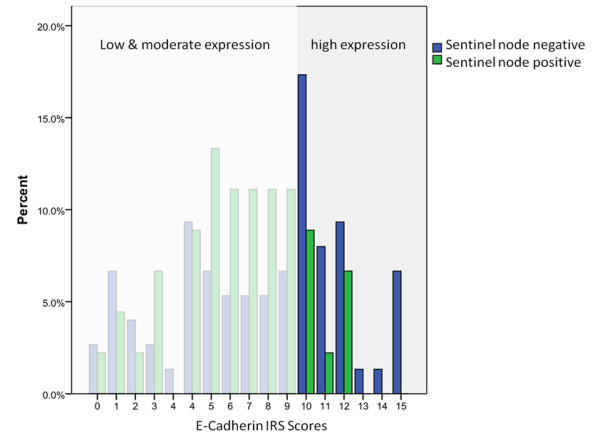
**ECAD high-expressors where predominantly sentinel node negative**.

High ECAD expression was found to correlate with negative lymph node status (*P *= 0.005) (table 2, figure 3). Even after controlling for tumour stage, ECAD expression remained a significant predictor for lymph node status (OR = 3.55, 95% CI: 1.450-8.694, *P *= 0.006; table 3).

**Table 3:** Adjusted odds ratios (ORs), 95% confidence intervals(95% CIs) and p-value of E-cadherin expression and tumour stage as predictors for lymph node status.

Predictor	OR	95% CI	*P*-value
Cadherin expression			
low vs. high	3.550	1.450-8.694	0.006
Tumour stage			
pT1 vs. pT2	0.590	0.272-1.278	0.181

Logistic regression: Adjusted odds ratios (OR), 95% confidence intervals (95% CI) and *p*-value of cadherin expression and tumor stage as predictors for lymph node status. Because of quasi-complete separation in the data, differentiation grade was not included as a factor in the logistic regression model.

To assess the diagnostic accuracy of ECAD expression as a predictive marker for SLN metastasis, sensitivity, specificity, negative and positive predictive values were determined. ECAD expression showed a sensitivity of 82.2% (95% CI: 0.674-0.915) and a specificity of 44% (95% CI: 0.327-0.559) with respect to presence and absence, respectively, of lymph node metastasis.

The positive predictive value was 66% and the negative predictive value was found to be 34.2%.

ECAD expression was also tested for a possible effect on relapse rates. The log rank test however did not indicate any influence of ECAD expression on relapse rates (*P *= 0.105).

## Discussion

In this study, we have confirmed our hypothesis and demonstrated that downregulation of cancer cell-expressed ECAD is associated with occult metastasis in oral cavity and oropharyngeal squamous cell carcinomas. These findings support the function of ECAD in tumour suppression and lymphogeneous metastasis in vivo.

In our study, approximately 90% of primary HNSCC express ECAD to some degree, but high expression levels have been shown to be significantly less associated with lymph node metastasis.

Our data are consistent with other recent studies, reporting low ECAD expression in squamous cell carcinomas of different organs to be correlated with invasion and metastasis and worse outcome [18-23]. These findings suggest that ECAD expression prohibits tumour disintegration and metastasis. However, the exact molecular function of cancer cell-expressed ECAD is currently studied.

Along with down regulation of ECAD, a phenomenon called Cadherin switching [24] namely the up regulation of other members of the Cadherin family like N-Cadherin or P-Cadherin has been found, which is also associated with worse outcome.

The problem of many study designs investigating predictive markers is the heterogeneity of primary tumours (location and T-stage) and N-stage which are lumped together. Since patients qualifying for a sentinel node biopsy yield a highly selected cohort (regarding tumour location and N-Status) with no clinical evidence of lymph node disease, we have as a consequence the possibility to detect cancers with occult metastatic disease at one of its earliest time points. Since we know that an existing tumour dedifferentiates with time and more advanced state, the possibility to find markers responsible for a metastatic phenotype is most promising in a study setting like ours. In this exceptionally well characterized and homogeneous cohort, we were able to study the process of early lymphatic metastasis and demonstrate a significant association between cancer cell-expressed ECAD and lymph node metastasis.

In the future, the combination of molecular markers with proven predictive value for lymphatic disease like ECAD, podoplanin [16,25], p16, bmi-1 [26], LOX [27] and histopathological features as mentioned above, could allow an individual risk stratification with possible impact on treatment strategy.

Sentinel node biopsy has been successfully validated for early HNSCC. However, use of SNB alone or SNB assisted elective neck dissection is still controversially discussed for staging and treatment of the cN0 neck in patients with early HNSCC. Additional biomarkers may be of help for better stratification of patients selected for SNB in contrast to elective neck dissection. The hitherto published predictive factors for metastatic disease in SNB include histomorphological parameters of the primary tumour like mode of invasion (MOI; morphological appearance of the infiltrating tumour front), grade of differentiation (GOD), lymphatic invasion (LI) [5] and intratumoural lymphatic density [6].

ECAD is a potential additional marker, because down-regulation of ECAD indicates an increased risk for lymph node metastasis. In the future, a combination of molecular markers like ECAD expression, and histomorphologic parameters may be used to stratify patients according to their risk for occult nodal disease.

## Conclusion

In summary, we provide evidence that downregulation of cancer cell-expressed ECAD correlates with higher incidence of lymph node metastasis in early HNSCC of the oral cavity and oropharynx. While ECAD might not be applicable as a predictive marker in a single test, ECAD immunohistochemistry might contribute among other factors to select patients for either elective neck dissection, sentinel lymph node biopsy or wait-and-watch policy.

## Competing interests

The authors declare that they have no competing interests.

## Authors' contributions

GFH, GS, HM and SJS participated in the design of the study and reviewed the final version of the manuscript. LZ was involved in the Tissue Micro Array production. LZ, AS and MR reviewed and scored the Immunohistochemistry of the cores. SKH and NG performed the statistical analysis. WJ provided and reviewed the paraffin blocks of patients from Kantonsspital St. Gallen. All authors read and approved the final manuscript.

## Pre-publication history

The pre-publication history for this paper can be accessed here:

http://www.biomedcentral.com/1471-2407/11/217/prepub
